# Radiomics Nomogram Based on Radiomics Score from Multiregional Diffusion-Weighted MRI and Clinical Factors for Evaluating HER-2 2+ Status of Breast Cancer

**DOI:** 10.3390/diagnostics11081491

**Published:** 2021-08-18

**Authors:** Chunli Li, Jiandong Yin

**Affiliations:** 1 Department of Radiology, Shengjing Hospital of China Medical University, Shenyang 110004, China; 2019121128@stu.cmu.edu.cn; 2 School of Intelligent Medicine, China Medical University, Shenyang 110122, China

**Keywords:** breast cancer, radiomics, diffusion-weighted imaging, HER-2

## Abstract

This study aimed to establish and validate a radiomics nomogram using the radiomics score (rad-score) based on multiregional diffusion-weighted imaging (DWI) and apparent diffusion coefficient (ADC) features combined with clinical factors for evaluating HER-2 2+ status of breast cancer. A total of 223 patients were retrospectively included. Radiomic features were extracted from multiregional DWI and ADC images. Based on the intratumoral, peritumoral, and combined regions, three rad-scores were calculated using the logistic regression model. Independent parameters were selected among clinical factors and combined rad-score (com-rad-score) using multivariate logistic analysis and used to construct a radiomics nomogram. The performance of the nomogram was evaluated using calibration, discrimination, and clinical usefulness. The areas under the receiver operator characteristic curve (AUCs) of intratumoral and peritumoral rad-scores were 0.824/0.763 and 0.794/0.731 in the training and validation cohorts, respectively. Com-rad-score achieved the highest AUC (0.860/0.790) among three rad-scores. ER status and com-rad-score were selected to establish the nomogram, which yielded good discrimination (AUC: 0.883/0.848) and calibration. Decision curve analysis demonstrated the clinical value of the nomogram in the validation cohort. In conclusion, radiomics nomogram, including clinical factors and com-rad-score, showed favorable performance for evaluating HER-2 2+ status in breast cancer.

## 1. Introduction

Female breast cancer (BC) ranks first in incidence globally and fifth as cause of cancer mortality worldwide [1]. BC is a heterogeneous tumor that is categorized into four major molecular subtypes: luminal A, luminal B, positive HER-2, and basal like [2]. Tumors with positive HER-2 account for approximately 20–30% of BCs and are associated with poor prognosis in the absence of systemic therapy [3]. HER-2-targeted therapy using anti-HER-2 antibodies (trastuzumab and pertuzumab) and small molecule tyrosine kinase inhibitors (lapatinib and neratinib) has beneficial effects on patients with positive HER-2 [4]. Therefore, accurate evaluation of HER-2 status is important for treatment decisions. Immunohistochemistry (IHC) is frequently performed to detect the expression of the HER-2 protein because it is easy to perform and cost-effective [5]. According to IHC results, a HER-2 staining intensity score of 3+ is considered positive, whereas a score of 0 or 1+ is considered negative. However, IHC cannot identify HER-2 2+ status, which accounted for approximately 17% of HER-2 patients [6], and a fluorescence in-situ hybridization (FISH) assay is required. Identification of gene amplification via FISH classifies tumors into positive or negative according to the HER-2 2+ status. However, this method of examination is cost-prohibitive, time consuming, and requires specialized equipment and technical skills. Therefore, there is an urgent need to establish a model to evaluate HER-2 2+ status.

Dynamic contrast-enhanced magnetic resonance imaging is a sensitive examination for the detection of BC [7]. However, owing to the recent concerns about the safety of gadolinium-including contrast agents, diffusion-weighted imaging (DWI) with apparent diffusion coefficient (ADC) maps has received increasing attention and is routinely used in a multiparametric imaging setting for BC detection [8,9,10,11,12]. It can provide functional information of tissue microstructure by measuring the random Brownian motion of water molecules and does not require intravenous injection of contrast agent [13]. Kim et al. showed that the ADC difference value derived from whole-lesion histogram analysis can be used to assess the recurrence risk in invasive BC patients with positive estrogen receptor (ER), negative HER-2, and negative node disease [14]. Another study demonstrated that maximum whole tumor ADC values may be used to discriminate luminal from other molecular subtypes of BC [15]. Radiomics transmutes medical images into high dimensional, mineable data using the technology for high-throughput extraction of quantitative features, and these data can be analyzed for decision support [16]. Therefore, this method may offer useful information about tumor heterogeneity. The radiomics model based on ADC maps achieved a good prediction performance for the Ki-67 status in patients with invasive ductal BC [17]. However, studies based on DWI for the diagnosis of BC have focused on investigating the predictive performance of imaging features within the tumor, ignoring the surrounding tissues. The peritumoral region may contain valuable information for the diagnosis of BC, such as peritumoral lymphatic vessel invasion, peritumoral lymphocytic infiltration, peritumoral edema, and peritumoral parenchyma [18,19,20,21]. Fan et al. showed that the ADC of the proximal peritumoral stroma could discriminate low from high Ki-67 groups in BC with positive ER [21]. A recent study found that the radiomic features from DWI and ADC images can be used to improve the performance for predicting different stages of rectal cancer [22]. Thus, the radiomic features from multiregional DWI and ADC images might be useful for evaluating HER-2 2+ status in patients with BC.

Guo et al. developed a nomogram including ER status, progesterone receptor (PR) status, and tumor grade to assess HER-2 2+ status in BC patients and achieved an area under the receiver operating characteristic (ROC) curve (AUC) of 0.749 in the validation cohort [6]. The diagnostic performance of that model was relatively low. Therefore, improving the diagnostic performance of the model is necessary for the identification of HER-2 2+ status in BC patients. Recent studies showed that a radiomics model constructed by incorporating clinical factors and radiomics score (rad-score) based on radiomic features could improve the predictive ability [23,24]. However, the diagnostic performance of clinical factors and the rad-score based on multiregional DWI and ADC images for evaluating HER-2 2+ status in BC patients has not been thoroughly investigated.

The purpose of this study was to establish a radiomics nomogram using the rad-score calculated according to multiregional DWI and ADC features combined with clinical factors for the assessment of HER-2 2+ status in BC and to analyze the performance of the radiomics model from each region.

## 2. Materials and Methods

### 2.1. Patient Cohort

The Institutional Ethics Committee approved the retrospective study, which was performed in accordance with the ethical principles for medical research involving human subjects as described in the 1964 Helsinki Declaration and its later amendments. The requirement of informed consent was waived owing to the retrospective nature of this study.

The information of all patients who underwent a preoperative breast DWI scan between November 2017 and April 2021 and who met the inclusion and exclusion criteria was retrospectively collected by three radiologists from the picture archiving and communication system (PACS). The inclusion criteria were: (i) confirmed diagnosis of BC based on pathological assessment of biopsy specimens; (ii) DWI scan performed <1 month before surgery; (iii) HER-2 score of 2+ verified by IHC; and (iv) presence of a mass-like single tumor (facilitating the subsequent segmentation of breast tumors). The exclusion criteria were: (i) any treatment before MRI scan, such as radiotherapy, chemotherapy, or chemoradiotherapy; (ii) incomplete pathology data (HER-2 2+ status unknown); and (iii) insufficient MRI quality, as determined by consensus of three radiologists (e.g., motion artifacts). Finally, 223 women with HER-2 2+ status verified by FISH were selected for this study, and the median lesion area was 352 mm^2^ (range: 195–1512 mm^2^).

**Figure 1:**
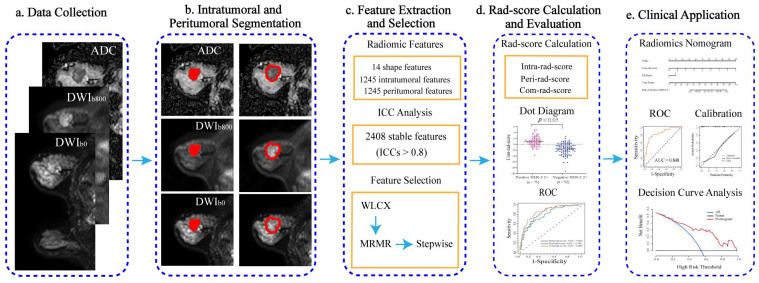
Flowchart of this study. (**a**) Image data (DWI_b=0_, DWI_b=800_, and ADC) was collected. (**b**) Intra- and peritumoral regions (red masks) were obtained using semi-automatic segmentation. (**c**) A total of 2504 radiomic features were extracted from three images, and ICC analysis and three-step feature selection were performed. (**d**) Three rad-scores were calculated with the selected features using the logistic regression model and evaluated. (**e**) Independent parameters were selected among clinical factors and combined rad-score (com-rad-score) using multivariate logistic analysis and used to establish a radiomics nomogram. ICC, intraclass correlation coefficient; WLCX, Wilcoxon rank-sum test; MRMR, minimum redundancy maximum relevance; ROC, receiver operating characteristic.

According to the time of treatment, all patients were divided into two cohorts at a ratio of 3:1. The training cohort included 167 patients (75 positive and 92 negative HER-2 2+) who received treatment between November 2017 and February 2020. The validation cohort included 56 patients (22 positive and 34 negative HER-2 2+) who received treatment between March 2020 and April 2021. The flowchart of this study is provided in Figure 1.

### 2.2. MRI Acquisition

All breast MRI examinations were performed with the patient in the prone position using a Signa HDxt 3.0 T MRI system (GE Healthcare Life Sciences, Chicago, IL, USA) with a dedicated eight-channel bilateral breast coil. An axial DWI sequence was performed for all patients. The acquisition parameters were as follows: repetition time, 4000 ms; echo time, 83.30 ms; flip angle, 90°; matrix size, 256 × 256; field of view, 340 m × 340 m; slices, 32; slice thickness, 4.50 mm; spacing between slices, 5 mm; and *b* values, 0 and 800 s/mm^2^.

All DWI (*b* = 0 and 800) data were exported from the PACS at the institution. ADC maps were calculated based on a pixel-by-pixel basis using the following formula: ADC = (lnSI_0_ − lnSI)/(*b* − *b*_0_), where SI_0_ and SI represent signal intensity obtained with *b* values of 0 and 800 s/mm^2^, respectively.

### 2.3. Tumor Segmentation

The slice image with largest tumor cross-section on DWI_b800_ was selected with evaluation consensus between two radiologists (Reader 1, with 10 years of experience in breast image interpretation, and Reader 2, with 5 years of experience). When any divergence of evaluation views existed, another senior radiologist (Reader 3, with 14 years of experience) was asked to make the final evaluation. Evaluators were blinded to the clinical and histopathological data.

Tumor segmentation was performed on the DWI_b800_ slice image selected above using MATLAB 2018a (Mathworks, Natick, MA, USA) by a radiologist (Reader 1). The intratumoral region of interest (ROI) was obtained using the following steps, as described in a previous study [25]:

First, an arbitrary shaped ROI was drawn around the lesion area.

Second, the maximum between-cluster variance method was applied to the ROI voxels, and the segmented image was converted into a binary image with the objective region as 1 and the background region as 0.

Third, morphological erosion was applied to the obtained binary image, and the size of the structural element was set at 4 × 4.

Fourth, a post-eroded image was traversed to obtain the largest unique eight-connected region.

Finally, morphological dilation of the unique region was performed, and the target region was considered as the intratumoral ROI.

The peritumoral ROI was obtained by dilating a distance of 4 mm from the boundary of the intratumoral ROI [26].

For tumors near the edge of the breast or chest wall, a breast parenchyma ROI (mask) was manually created using itk-SNAP software (version 3.6.0, Philadelphia, PA, USA) and loaded into MATLAB 2018a [27]. The peritumoral ROI was additionally bounded to the breast tissue ROI so that the peritumoral ROI was not outside of the breast region [27].

The contours of intra- and peritumoral ROIs on DWI_b800_ were copied to the exact same location of the corresponding DWI_b0_ and ADC images. Then, the segmentation results of ADC and DWI_b0_ were validated by Reader 1.

### 2.4. Radiomic Feature Extraction

Image intensity normalization and feature extraction were performed using MATLAB 2018a (Mathworks, Natick, MA, USA). Prior to feature extraction, all pixel intensities within the intra- and peritumoral ROIs were normalized between μ ± 3σ (μ, mean of image intensity within the ROI; σ, standard deviation), and the gray level range was quantized to eight bits/pixel [28].

Five categories of features were extracted from three images (DWI_b0_, DWI_b800_, and ADC) according to previous radiomic studies [26,29,30,31]: (i) shape features, (ii) first-order statistics features, (iii) gray-level co-occurrence matrix (GLCM) features, (iv) Laws features, and (v) Gabor features. Detailed descriptions of these radiomic features are provided in Table 1. All features were then normalized to z distribution ((value−mean value)/standard deviation).

### 2.5. Interobserver Variability Evaluation

Sixty images, including 30 positive and 30 negative HER-2 2+, were randomly selected for ROI segmentation by two radiologists (Reader 1 and Reader 2). Then, radiomic features were extracted from the segmented images of each radiologist. Intraclass correlation coefficient (ICC) analysis was performed to evaluate the reproducibility and stability of radiomic feature extraction. Features with an ICC > 0.8 were considered to have good agreement and selected for subsequent radiomics analysis [32,33].

### 2.6. Feature Selection and Radiomics Score Calculation

A three-step feature selection was conducted based on the training cohort. First, the features with *p* < 0.1 between the positive and negative HER-2 2+ groups were identified using Wilcoxon rank-sum test (WLCX). Second, 20 features with high relevance and low redundancy were chosen by applying minimum redundancy maximum relevance (MRMR). Third, the optimal features were selected based on the backward stepwise method where the stopping rule was set to the likelihood ratio test with Akaike’s information criterion [34].

Feature selection for the intratumoral, peritumoral, and combined regions was performed according to the HER-2 2+ status. Next, the intratumoral rad-score (intra-rad-score), peritumoral rad-score (peri-rad-score), and combined rad-score (com-rad-score) were calculated using the logistic regression model [34]. The AUC was used to evaluate the discriminative performance of three rad-scores in the training and validation cohorts.

### 2.7. Radiomics Nomogram Establishment

The potential predictors were first identified among clinical factors and com-rad-score using univariate logistic regression. These predictors were fed into the multivariate logistic regression, which was used to select independent predictors of positive HER-2 2+. A radiomics nomogram was established based on the independent predictors. The discrimination performances of the radiomics nomogram in the training and validation cohorts were assessed using the AUCs. The calibration curve was generated to analyze the agreement between the observed and predicted risks of positive HER-2 2+, and the calibration performances of the radiomics nomogram were assessed in the training and validation cohorts. The clinical usefulness of the radiomics nomogram was evaluated in the validation cohort using decision curve analysis (DCA).

### 2.8. Statistical Analysis

Differences in categorical variables between positive and negative HER-2 2+ groups were analyzed using the chi-square test. The independent sample *t*-test was used to investigate the associations between rad-scores and HER-2 2+ status. A *p* value < 0.05 was considered statistically significant. The DeLong test was used to statistically compare the AUC values between two models. The statistical analyses, feature selection, model construction, and figure plots were performed using R software (version 3.6.2).

## 3. Results

### 3.1. Patient Characteristics

**Figure 2:**
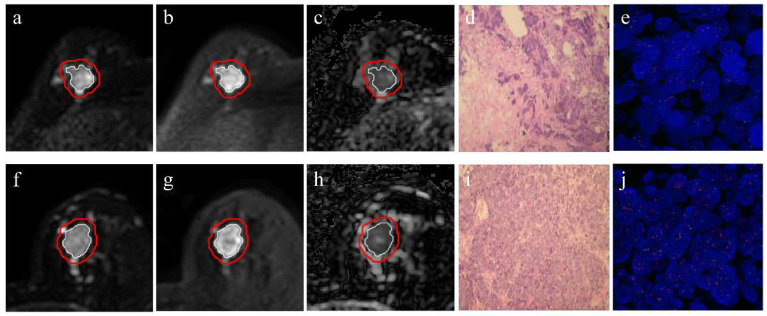
Intratumoral segmentation, peritumoral segmentation, pathology, and FISH results of randomly selected cases with negative and positive HER-2 2+. Case with negative HER-2 2+: (**a**) DWI_b=0_, (**b**) DWI_b=800_, (**c**) ADC map, (**d**) pathology findings, and (**e**) FISH results. Case with positive HER-2 2+: (**f**) DWI_b=0_, (**g**) DWI_b=800_, (**h**) ADC map, (**i**) pathology findings, and (**j**) FISH results. The white and red lines indicate the intratumoral and peritumoral margins on DWI_b=0_, DWI_b=800_, and ADC images, respectively.

The patient characteristics are shown in Table 2. No significant difference in the rates of positive HER-2 2+ was observed between the two cohorts (75/167, 44.91% vs. 22/56, 39.29%, *p* = 0.463). There were significant differences in ER (*p* < 0.001), PR (*p* < 0.001), and Ki-67 (*p* = 0.002) status between the positive and negative HER-2 2+ groups but not in age (*p* = 0.866). Two randomly selected cases are provided in Figure 2 to display the results of intratumoral segmentation, peritumoral segmentation, pathology, and FISH.

### 3.2. Feature Selection, Rad-Score Calculation, and Evaluation

Of the 2504 extracted radiomic features, 2408 (96.17%) had good interobserver agreement with ICCs > 0.8, including 14 shape, 1210 intratumor, and 1184 peritumor features.

Four Laws and five Gabor features were selected to calculate the intra-rad-score. Five Laws and four Gabor features were chosen to calculate the peri-rad-score. Seven Laws and six Gabor features were selected to calculate the com-rad-score. The features respectively derived from DWI_b0_, DWI_b800_, and ADC images could be observed among the selected features used for each rad-score calculation (intra-rad-score: 3/2/4; peri-rad-score: 2/3/4; com-rad-score: 5/3/5). Further details about the features and rad-score calculation formulas are provided in Supplementary Table S1.

**Figure 3:**
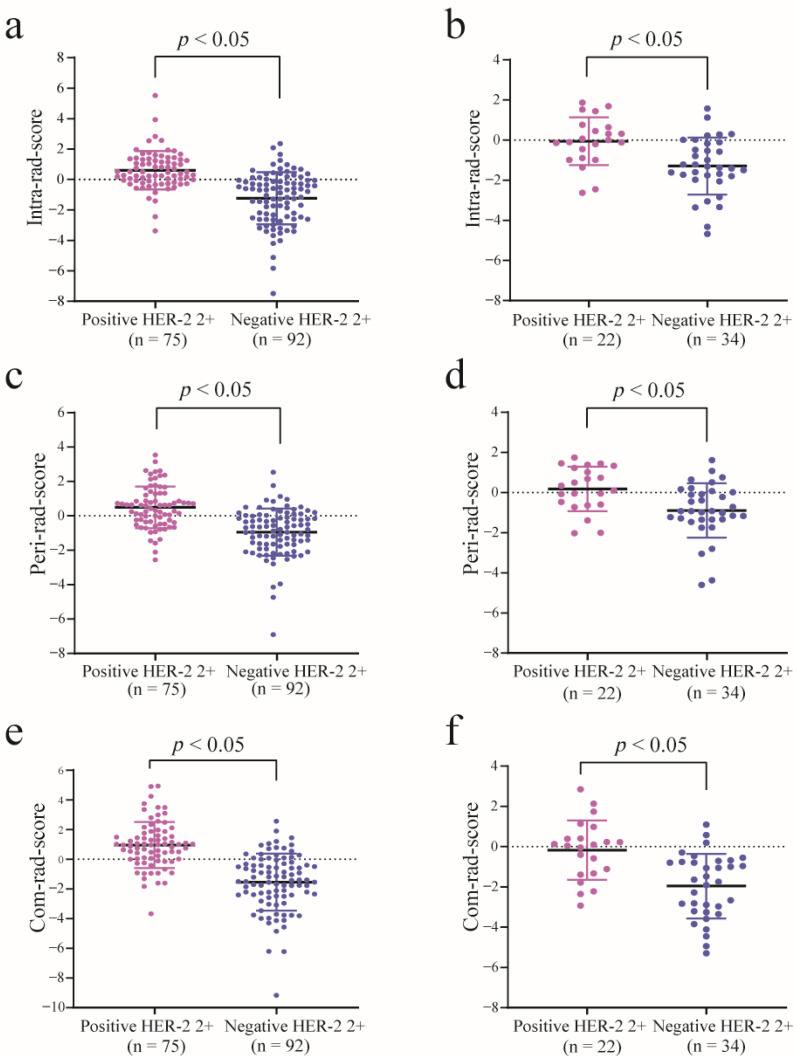
Dot diagram of the three rad-scores in the training (positive: *n* = 75, negative: *n* = 92) and validation (positive: *n* = 22, negative: *n* = 34) cohorts. Dot diagram of the intra-rad-score in the training (**a**) and validation (**b**) cohorts. Dot diagram of the peri-rad-score in the training (**c**) and validation (**d**) cohorts. Dot diagram of the com-rad-score in the training (**e**) and validation (**f**) cohorts.

**Figure 4:**
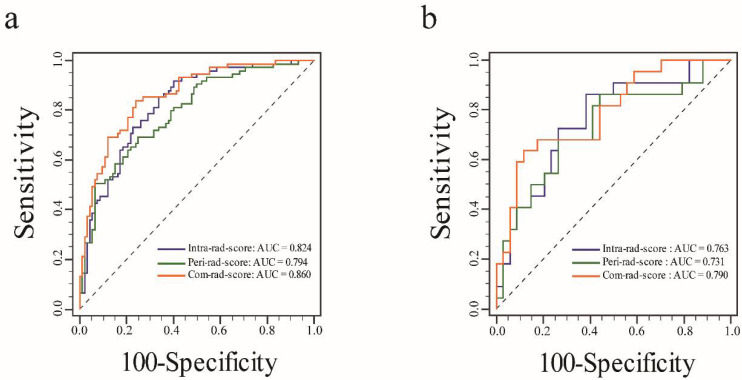
ROC curves of the three rad-scores in each cohort. (**a**) ROC curves of the three rad-scores in the training cohort. (**b**) ROC curves of the three rad-scores in the validation cohort.

There were significant differences in intra-, peri-, and com-rad-scores between the positive and negative HER-2 2+ groups in the training cohort (all *p* < 0.05) and in the validation cohort (all *p* < 0.05). Patients with positive HER-2 2+ showed generally higher values in the three rad-scores (Figure 3). The ROC curves of three rad-scores are shown in Figure 4. Discriminative performances of three rad-scores in the training and validation cohorts are shown in Table 3. The com-rad-score achieved the highest AUC (0.860) among the three rad-scores in the training cohort, and the same result (0.790) was obtained in the validation cohort. The intra-rad-score obtained an AUC of 0.824 and 0.763 in the training cohort and validation cohort, respectively. The AUC of the peri-rad-score was 0.794 and 0.731 in the training cohort and validation cohort, respectively. No significant differences of AUCs between the intra-rad-score and peri-rad-score (*p* = 0.476) and between the intra-rad-score and com-rad-score (*p* = 0.182), were observed in the training cohort. However, significant difference existed between the peri-rad-score and com-rad-score (*p* = 0.031). In addition, no significant differences (intra-rad-score vs. peri-rad-score, *p* = 0.681; intra-rad-score vs. com-rad-score, *p* = 0.637; and peri-rad-score vs. com-rad-score, *p* = 0.370) were presented in the validation cohort. Detailed results are provided in Table 4.

### 3.3. Radiomics Nomogram Establishment and Assessment

**Figure 5:**
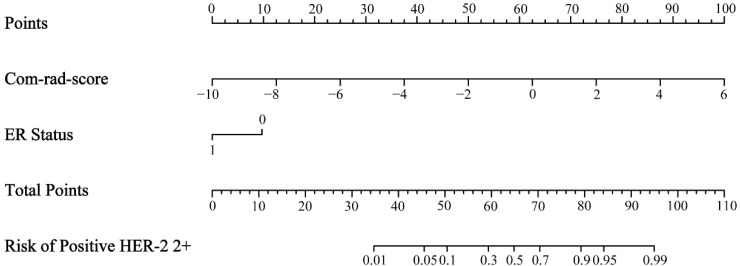
Radiomics nomogram incorporating the com-rad-score and ER status.

**Figure 6:**
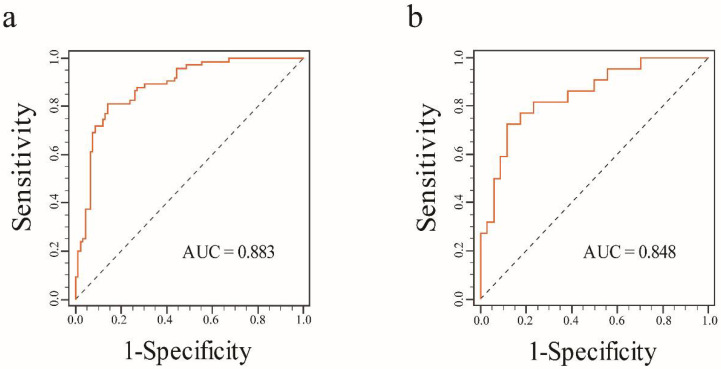
ROC curves for the radiomics nomogram in each cohort. (**a**) ROC curve for the radiomics nomogram in the training cohort. (**b**) ROC curve for the radiomics nomogram in the validation cohort.

**Figure 7:**
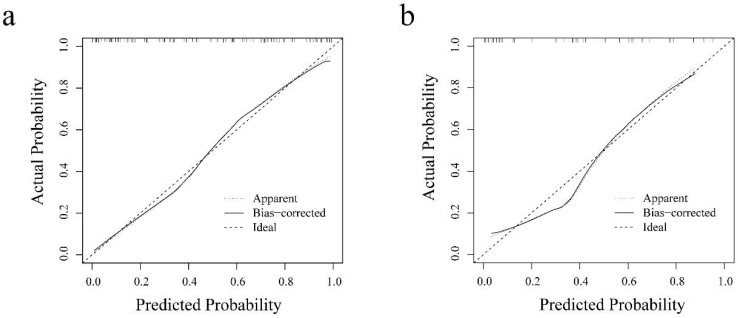
Calibration curves of the radiomics nomogram in each cohort. (**a**) Calibration curve of the radiomics nomogram in the training cohort. (**b**) Calibration curve of the radiomics nomogram in the validation cohort.

**Figure 8:**
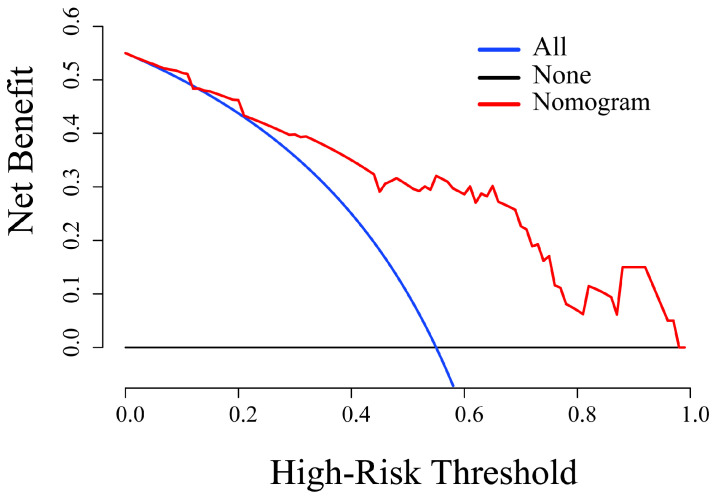
DCA for the radiomics nomogram in the validation cohort.

The results of univariate and multivariate logistic regression analyses are summarized in Table 5. The com-rad-score (odds ratio (OR): 2.644, confidence interval (CI): 1.888–3.702, *p* < 0.001) and ER status (OR: 8.255, CI: 1.745–39.044, *p* = 0.008) were selected as independent predictors. Next, a radiomics nomogram was developed including the com-rad-score and ER status (Figure 5). The ROC curves of the radiomics nomogram are provided in Figure 6. Discriminative performances of radiomics nomogram in the training and validation cohorts are shown in Table 4. The radiomics nomogram yielded an AUC of 0.883 and 0.848 in the training and validation cohorts, respectively. No significant differences of AUCs between the radiomics nomogram and intra-rad-score (*p* = 0.053) and between the radiomics nomogram and com-rad-score (*p* = 0.068) were observed in the training cohort. However, significant difference existed between the radiomics nomogram and peri-rad-score (*p* = 0.002). Moreover, no significant differences (radiomics nomogram vs. intra-rad-score, *p* = 0.162; radiomics nomogram vs. peri-rad-score, *p* = 0.082; and radiomics nomogram vs. com-rad-score, *p* = 0.096) were observed in the validation cohort. Detailed results are shown in Table 4. The calibration curves of the radiomics nomogram are shown in Figure 7. The calibration curves indicated that the radiomics nomogram had the ability of good calibration in the training and validation cohorts. The results of the DCA for the radiomics nomogram are presented in Figure 8. The DCA indicated that applying the radiomics nomogram was more beneficial than the treat-all strategy and the treat-none strategy when the range of threshold probability was >21%.

## 4. Discussion

In this study, three rad-scores were first calculated according to the intratumoral, peritumoral, and multiregional features in DWI and ADC images. The ROC curves showed that the com-rad-score based on multiregional features achieved the highest AUC in both the training and validation cohorts. A radiomics nomogram was then established using com-rad-score and ER status. The results indicated that the radiomics nomogram yielded good discrimination and calibration.

Studies have investigated the diagnostic value of imaging features from ADC maps for assessing the genetic status of BC [15,35]. However, these studies only evaluated the performance of histogram features in intratumoral regions. Advances in radiomics have provided more high-order radiomic features that can be analyzed to comprehensively describe tumor heterogeneity. Zhang et al. constructed an ADC-based radiomics model using 11 radiomic features (selected from 1029 extracted features) in intratumor regions for predicting Ki-67 status in patients with invasive ductal BC, and this model showed good diagnostic ability. However, these studies mainly analyzed the features from intratumoral regions [17]. BC involves not only neoplastic cells but also significant alterations in the surrounding stroma or tumor microenvironment [36]. A recent study found the differences on ADC maps of the proximal peritumoral stroma between high and low Ki-67 in BC patients with positive ER [21]. In our study, low- and high-order radiomic features from intra- and peritumoral regions of DWI and ADC images were extracted to improve the performance of the model. Then, intra- and peri-rad-scores were calculated according to the features selected from the corresponding regions. The results indicated that the intra-rad-score and peri-rad-score had the potential value for the evaluation of HER-2 2+ status. A com-rad-score was also calculated by combining the intra- and peritumoral features in this study. The ROC curve indicated that the com-rad-score yielded a higher AUC score than intra- and peri-rad-scores in both the training and validation cohorts. The results were in agreement with those of several recent studies, which indicated that a radiomics model including intra- and peritumoral features could improve the diagnostic performance for predicting the pathological outcome in BC [29,30,37].

The reproducibility and robustness of radiomic feature extraction performed by two radiologists were determined by calculating ICCs. The results demonstrated that most of the features were in good agreement. After feature selection with WLCX, MRMR, and stepwise, the radiomic features obtained from each modality image were found among the finally selected features for the calculation of three rad-scores, which demonstrated that three images, DWI_b0_, DWI_b800_, and ADC, were all essential and could offer complementary information for the detection of HER-2 2+ status. The features used to calculate the three rad-scores were mainly Law and Gabor features, which may reflect intra- and peritumor heterogeneity. Gabor features can detect the wavelike patterns of intensity variation across different spatial scales in different orientations, and Law features can capture the patterns of inconsistent enhancement and abnormal structure [26,29,31]. However, detailed intra- and peritumor features are usually difficult to detect using the naked eye, whereas they can be easily detected by radiomics analysis.

In this study, ER status and com-rad-score were identified as independent predictors using univariate and multivariate logistic regression analyses. Positive HER-2 2+ correlated significantly with negative ER status (Figure 5). This result was consistent with that of a previous study [38]. A radiomics nomogram was established incorporating ER status and com-rad-score. The nomogram yielded an AUC of 0.848 in the validation cohort, which was higher than that of previous nomograms for HER-2 2+ status determination that only used clinicopathologic characteristics of BC (AUC: 0.749) [6]. The nomogram also achieved a higher AUC than the com-rad-score. This finding indicated that the diagnostic performance of the radiomics model by combining the com-rad-score with clinical factors could be improved. Several recent studies reported similar findings that further support the value of the radiomics model established using rad-score and clinical factors for evaluating pathological outcomes [26,32,39]. Finally, DCA demonstrated the feasibility of the established nomogram for clinical application.

There are several limitations to our study. First, the retrospective nature of this study may have led to potential selection bias. Second, the number of patients included in the study was limited, and patients were from a single center. Therefore, multicenter studies with a larger number of patients are needed to validate the performance of the constructed models. Third, only two-dimensional images with the largest tumor cross-section were used for the radiomics analysis. This could lead to missed information because of the heterogeneity of tumor volume. A radiomics model based on three-dimensional segmentation should be developed in future studies. Finally, more and more studies on BC diagnosis were performed based on deep neural learning, which was a subset of machine learning and unsupervised from data that were unstructured and unlabeled [40,41,42,43]. In our study, only conventional radiomics analysis was investigated, and the difference of performance and robustness in evaluating HER-2 2+ status should be further compared between our study and those based on deep neural network.

## 5. Conclusions

The radiomics nomogram incorporating ER status and com-rad-score showed a favorable performance for predicting HER-2 2+ status in patients with BC. Therefore, it could be used as a supplementary method. An external validation cohort consisting of a large number of samples is necessary to evaluate the effectiveness of the established nomogram before clinical application.

## Figures and Tables

**Table 1:** List of Radiomic Features.

Category (Quantity)	Radiomic Features
Shape *n* = 14 ^a^	Area, Perimeter, Sphericity, Elongation, Extent, Circularity, Solidity, Eccentricity, Equivalent diameter, Major axis length, Minor axis length, Perimeter to area ratio, Maximum 2D diameter, Spherical disproportion.
First-order statistics *n* = 5 ^b^	Mean, Median, SD, Skewness, Kurtosis.
GLCM *n* = 45 ^c^	Energy, Contrast, Correlation, Variance, Entropy, Homogeneity, Inverse difference moment, Information measures of correlation 1, Information measures of correlation 2.
Laws *n* = 125 ^c^	Response to 5-pixel × 5-pixel filter targeting the specific texture enhancement patterns in the X and Y directions. 25 descriptors are derived from all combinations of five one-dimensional filters: level (L), edge (E), spot (S), wave (W), and ripple (R). L5 = (1 4 6 4 1), E5 = (−1 −2 0 2 1), S5 = (−1 0 2 0 −1), R5 = (1 −4 6 −4 1), and W5 = (−1 2 0 −2 −1). The 25 filters were L5L5, L5E5, L5S5, L5W5, L5R5, E5L5, E5E5, E5S5, E5W5, E5R5, S5L5, S5E5, S5S5, S5W5, S5R5, W5L5, W5E5, W5S5, W5W5, W5R5, R5L5, R5E5, R5S5, R5W5, and R5R5.
Gabor *n* = 240 ^c^	Gabor wavelet is sensitive to image edge and has good spatial locality and directional selectivity and can grasp the spatial frequency (scale) and local structure characteristics of multiple directions in the local area of the image. Each descriptor quantifies response to a given Gabor filter at a specific frequency (f = 0, 2, 4, 8, 16, 32) and orientation (θ = 0°, 22.5°, 45°, 67.5°, 90°, 112.5°, 135°, 167.5°).

^a^ Shape features were extracted from the intratumoral ROIs. ^b^ First-order statistics for three original images (DWI_b0_, DWI_b800_, and ADC) within the intra- and peritumoral ROIs were calculated. ^c^ First-order statistics (mean, median, SD, skewness, and kurtosis) for per descriptor per image per ROI were calculated (three images, both intratumoral and peritumoral ROIs).

**Table 2:** Characteristics of 223 patients with HER-2 2+ BC.

	FISH Results		
Characteristics	Positive HER-2 2+ (*n* = 97, 43.50%)	Negative HER-2 2+ (*n* = 126, 56.50%)	*p* Value
Age (%)			0.866
<40 years	17 (17.53)	21 (16.67)	
≥40 years	80 (82.47)	105 (83.33)	
ER status (%)			<0.001 *
Negative	39 (40.21)	14 (11.11)	
Positive	58 (59.79)	112 (88.89)	
PR status (%)			<0.001 *
Negative	38 (39.18)	21 (16.67)	
Positive	59 (60.82)	105 (83.33)	
Ki-67 (%)			0.002 *
<14%	17 (17.53)	46 (36.51)	
≥14%	80 (82.47)	80 (63.49)	

The chi-square test was used to compare the differences in categorical variables between the positive and negative HER-2 groups. * *p* value < 0.05 was considered statistically significant.

**Table 3:** Discriminative performances of three rad-scores and radiomics nomogram for predicting HER-2 2+ status in the training and validation cohorts.

Cohort	Intra-Rad-Score	Peri-Rad-Score	Com-Rad-Score	Radiomics Nomogram
Training cohort				
AUC (95% CI)	0.824 (0.769–0.884)	0.794 (0.726–0.850)	0.860 (0.824–0.925)	0.883 (0.844–0.938)
Sensitivity (95% CI)	0.920 (0.859–0.981)	0.693 (0.589–0.798)	0.840 (0.757–0.923)	0.813 (0.725–0.902)
Specificity (95% CI)	0.598 (0.498–0.698)	0.750 (0.662–0.838)	0.761 (0.674–0.848)	0.859 (0.788–0.930)
Accuracy (95% CI)	0.743 (0.740–0.745)	0.725 (0.722–0.727)	0.796 (0.795–0.798)	0.838 (0.837–0.840)
Validation cohort				
AUC (95% CI)	0.763 (0.631–0.867)	0.731 (0.596–0.841)	0.790 (0.661–0.887)	0.848 (0.726–0.930)
Sensitivity (95% CI)	0.864 (0.720–1.000)	0.864 (0.720–1.000)	0.636 (0.435–0.837)	0.727 (0.541–0.913)
Specificity (95% CI)	0.618 (0.454–0.781)	0.559 (0.392–0.726)	0.882 (0.774–0.991)	0.882 (0.774–0.991)
Accuracy (95% CI)	0.714 (0.707–0.721)	0.679 (0.671–0.686)	0.786 (0.780–0.792)	0.821 (0.816–0.827)

CI, confidence interval.

**Table 4:** The statistical comparison of AUC values using the DeLong test among four models.

Cohort	Models	Intra-Rad-Score	Peri-Rad-Score	Com-Rad-Score	Radiomics Nomogram
Training	Intra-rad-score	/	0.476	0.182	0.053
	Peri-rad-score	0.476	/	0.031	0.002
	Com-rad-score	0.182	0.031	/	0.068
	Radiomics nomogram	0.053	0.002	0.068	/
Validation	Intra-rad-score	/	0.681	0.637	0.162
	Peri-rad-score	0.681	/	0.370	0.082
	Com-rad-score	0.637	0.370	/	0.096
	Radiomics nomogram	0.162	0.082	0.096	/

The slash indicates that there is no data here.

**Table 5:** Univariate and multivariate logistic regression analyses of the clinical parameters and com-rad-score for HER-2 2+ status in the training cohort.

	Univariate Analysis		Multivariate Analysis	
Parameters	OR (95% CI)	*p* Value	OR (95% CI)	*p* Value
Age				
<40 years	1			
≥40 years	1.126 (0.538–2.359)	0.753		
ER status				
Negative	1		1	
Positive	5.480 (2.516–11.936)	<0.001 *	8.255 (1.745–39.044)	0.008 *
PR status				
Negative	1		1	
Positive	3.535 (1.743–7.169)	<0.001 *	0.422 (0.096–1.860)	0.254
Ki-67				
<14%	1		1	
≥14%	0.447 (0.220–0.908)	0.026 *	0.589 (0.227–1.528)	0.277
Com-rad-score	2.718 (1.975–3.741)	<0.001 *	2.644 (1.888–3.702)	<0.001 *

OR, odds ratio; CI, confidence internal. * *p* < 0.05 was considered statistically significant in the univariate logistic regression analysis. Then, those significant parameters were incorporated into the multivariate logistic regression analysis. Finally, the parameters with *p* < 0.05 were selected as independent predictors.

## Data Availability

The data that support the findings of this study are available on request from the corresponding author. The data are not publicly available due to privacy or ethical restrictions.

## Supplementary Materials

The following are available online at https://www.mdpi.com/article/10.3390/diagnostics11081491/s1, Table S1: Radiomic features identified in each Region to distinguish positive from negative HER-2 2+.
