# Supplementary material for: Radiomics Nomogram Based on Radiomics Score from Multiregional Diffusion-Weighted MRI and Clinical Factors for Evaluating HER-2 2+ Status of Breast Cancer
Source: Diagnostics (Basel). 2021 Aug 18;11(8):1491. doi: 10.3390/diagnostics11081491 (PMC8395031; doi:10.3390/diagnostics11081491)
Supplement: Supplementary file 1 [file diagnostics-11-01491-s001.zip › diagnostics-1316695-supplementary.pdf]

Table S1. Radiomic features identified in each Region to distinguish positive from negative HER-2 2+.

No.	Feature Family	Descriptor	Statistic	Image	Location	Coefficient in LR model
	Intratumoral radiomic features					
I1	Gabor	$f = 2, \theta = 22.5°$	Median	ADC	Intratumor	-0.366
I2	Gabor	$f = 32, \theta = 0°$	Kurtosis	DWI_{b0}	Intratumor	0.404
I3	Laws	R5S5	Skewness	DWI_{b0}	Intratumor	-0.755
I4	laws	E5L5	Skewness	ADC	Intratumor	-0.617
I5	Gabor	$f = 16, \theta = 0°$	Kurtosis	ADC	Intratumor	-0.387
I6	Laws	E5E5	Median	DWI_{b800}	Intratumor	-0.669
I7	Gabor	$f = 8, \theta = 0°$	Skewness	DWI_{b0}	Intratumor	0.578
I8	Laws	L5E5	Median	ADC	Intratumor	-0.708
I9	Gabor	$f = 32, \theta = 112.5°$	Kurtosis	DWI_{b800}	Intratumor	0.346
	Peritumoral radiomic features					
P1	Gabor	$f = 4, \theta = 67.5°$	Kurtosis	DWI_{b800}	Peritumor	0.484
P2	Laws	E5R5	Skewness	DWI_{b0}	Peritumor	-0.369
P3	Laws	W5W5	SD	DWI_{b800}	Peritumor	0.358
P4	Laws	R5W5	Mean	DWI_{b0}	Peritumor	0.334
P5	Gabor	$f = 8, \theta = 112.5°$	Median	ADC	Peritumor	0.373
P6	Gabor	$f = 32, \theta = 112.5°$	Kurtosis	DWI_{b800}	Peritumor	0.394
P7	Gabor	$f = 8, \theta = 90°$	Kurtosis	ADC	Peritumor	0.404
P8	Laws	L5E5	Median	ADC	Peritumor	-0.553
P9	Laws	E5L5	Skewness	ADC	Peritumor	-0.687
	Combined radiomic features					
C1	Gabor	$f = 4, \theta = 67.5°$	Kurtosis	DWI_{b800}	Peritumor	0.416
C2	Laws	E5R5	Skewness	DWI_{b0}	Peritumor	-0.540
C3	Laws	R5S5	Skewness	DWI_{b0}	Intratumor	-0.739

C4	Laws	E5L5	Skewness	ADC	Peritumor	-0.564
C5	Gabor	f = 16, θ = 0°	Kurtosis	ADC	Intratumor	-0.605
C6	Gabor	f = 32, θ = 0°	Kurtosis	DWI_{b0}	Peritumor	0.485
C7	Laws	W5W5	SD	DWI_{b800}	Peritumor	0.412
C8	Gabor	f = 16, θ = 45°	Median	ADC	Peritumor	0.405
C9	Laws	R5R5	SD	DWI_{b0}	Intratumor	0.373
C10	Gabor	f = 8, θ = 90°	Skewness	ADC	Peritumor	0.316
C11	Laws	E5E5	Median	DWI_{b800}	Intratumor	-0.584
C12	Gabor	f = 8, θ = 0°	Skewness	DWI_{b0}	Intratumor	0.643
C13	Laws	L5E5	Median	ADC	Intratumor	-0.650

Abbreviations: SD, standard deviation; LR, logistic regression.

Intra-rad-score, peri-rad-score, and com-rad-score calculation formulas in predicting HER-2 2+ status:

Intra-rad-score = - 0.506 - 0.366 × I1 + 0.404 × I2 - 0.755 × I3 - 0.617 × I4 - 0.387 × I5 - 0.669 × I6 + 0.578 × I7 - 0.708 × I8 + 0.346 × I9

Peri-rad-score = - 0.344 + 0.484 × P1 - 0.369 × P2 + 0.358 × P3 + 0.334 × P4 + 0.373 × P5 + 0.394 × P6 + 0.404 × P7 - 0.553 × P8 - 0.687 × P9

Com-rad-score = - 0.633 + 0.416 × C1 - 0.540 × C2 - 0.739 × C3 - 0.564 × C4 - 0.605 × C5 + 0.485 × C6 + 0.412 × C7 + 0.405 × C8 + 0.373 × C9 + 0.316 × C10 - 0.584 × C11 + 0.643 × C12 - 0.650 × C13
